# Imaging Glioblastoma Metabolism by Using Hyperpolarized [1-^13^C]Pyruvate Demonstrates Heterogeneity in Lactate Labeling: A Proof of Principle Study

**DOI:** 10.1148/rycan.210076

**Published:** 2022-07-15

**Authors:** Fulvio Zaccagna, Mary A. McLean, James T. Grist, Joshua Kaggie, Richard Mair, Frank Riemer, Ramona Woitek, Andrew B. Gill, Surrin Deen, Charlie J. Daniels, Stephan Ursprung, Rolf F. Schulte, Kieren Allinson, Anita Chhabra, Marie-Christine Laurent, Matthew Locke, Amy Frary, Sarah Hilborne, Ilse Patterson, Bruno D. Carmo, Rhys Slough, Ian Wilkinson, Bristi Basu, James Wason, Jonathan H. Gillard, Tomasz Matys, Colin Watts, Stephen J. Price, Thomas Santarius, Martin J. Graves, Sarah Jefferies, Kevin M. Brindle, Ferdia A. Gallagher

**Affiliations:** From the Departments of Radiology (F.Z., J.T.G., J.K., F.R., R.W., A.B.G., S.D., C.J.D., S.U., M.C.L., M.L., A.F., S.H., J.H.G., T.M., M.J.G., F.A.G.), Clinical Neurosciences (R.M., C.W., S.J.P., T.S.), and Medicine (I.W.), University of Cambridge School of Clinical Medicine, Cambridge, England; Cancer Research UK Cambridge Institute (M.A.M., S.U., K.M.B.), Medical Research Council Biostatistics Unit (J.W.), and Department of Biochemistry (K.M.B.), University of Cambridge, Li Ka Shing Centre, Robinson Way, Cambridge, CB2 0RE, England; Department of Biomedical Imaging and Image-guided Therapy, Medical University of Vienna, Vienna, Austria (R.W.); GE Healthcare, Munich, Germany (R.F.S.); Department of Pathology (K.A.), Cambridge Cancer Trials Centre (A.C.), Department of Radiology (I.P., B.D.C., R.S.), and Department of Oncology (B.B., S.J.), Cambridge University Hospitals National Health Service Foundation Trust, Cambridge, England; and Population Health Sciences Institute, Newcastle University, Newcastle upon Tyne, England (J.W.).

**Keywords:** Hyperpolarized ^13^C MRI, Glioblastoma, Metabolism, Cancer, MRI, Neuro-oncology

## Abstract

**Purpose:**

To evaluate glioblastoma (GBM) metabolism by using hyperpolarized carbon 13 (^13^C) MRI to monitor the exchange of the hyperpolarized ^13^C label between injected [1-^13^C]pyruvate and tumor lactate and bicarbonate.

**Materials and Methods:**

In this prospective study, seven treatment-naive patients (age [mean ± SD], 60 years ± 11; five men) with GBM were imaged at 3 T by using a dual-tuned ^13^C–hydrogen 1 head coil. Hyperpolarized [1-^13^C]pyruvate was injected, and signal was acquired by using a dynamic MRI spiral sequence. Metabolism was assessed within the tumor, in the normal-appearing brain parenchyma (NABP), and in healthy volunteers by using paired or unpaired *t* tests and a Wilcoxon signed rank test. The Spearman ρ correlation coefficient was used to correlate metabolite labeling with lactate dehydrogenase A (LDH-A) expression and some immunohistochemical markers. The Benjamini-Hochberg procedure was used to correct for multiple comparisons.

**Results:**

The bicarbonate-to-pyruvate (BP) ratio was lower in the tumor than in the contralateral NABP (*P* < .01). The tumor lactate-to-pyruvate (LP) ratio was not different from that in the NABP (*P* = .38). The LP and BP ratios in the NABP were higher than those observed previously in healthy volunteers (*P* < .05). Tumor lactate and bicarbonate signal intensities were strongly correlated with the pyruvate signal intensity (ρ = 0.92, *P* < .001, and ρ = 0.66, *P* < .001, respectively), and the LP ratio was weakly correlated with LDH-A expression in biopsy samples (ρ = 0.43, *P* = .04).

**Conclusion:**

Hyperpolarized ^13^C MRI demonstrated variation in lactate labeling in GBM, both within and between tumors. In contrast, bicarbonate labeling was consistently lower in tumors than in the surrounding NABP.

**Keywords:** Hyperpolarized ^13^C MRI, Glioblastoma, Metabolism, Cancer, MRI, Neuro-oncology

*Supplemental material is available for this article.*

Published under a CC BY 4.0 license.

## Graphical abstract

**Figure:**
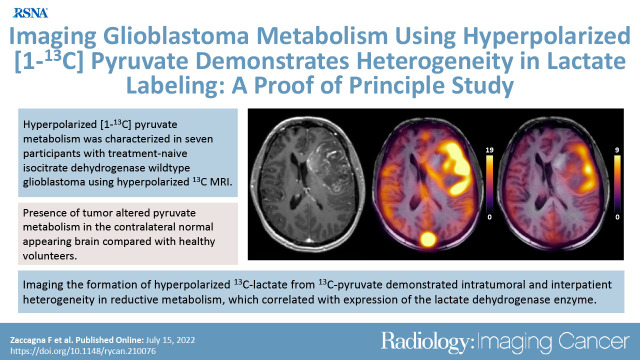

**Summary**

Carbon 13 (^13^C) MRI following injection of hyperpolarized ^13^C-labeled pyruvate can be used to characterize glioblastoma metabolism and changes in the surrounding brain parenchyma, and this tumor metabolism may be correlated with metabolic gene expression.

**Key Points**

■ Imaging the formation of hyperpolarized carbon 13 (^13^C)-lactate from ^13^C-pyruvate demonstrated intratumoral and interpatient heterogeneity in reductive metabolism, and the lactate-to-pyruvate (LP) ratio correlated with expression of the enzyme lactate dehydrogenase A (*P* = .04).
■ The hyperpolarized ^13^C-bicarbonate signal intensity was consistently reduced in glioblastoma (GBM) compared with in the contralateral brain (bicarbonate-to-pyruvate [BP] ratio, 0.06 ± 0.03 vs 0.10 ± 0.03, *P* = .002), consistent with a reduction in oxidative metabolism.
■ The presence of tumor altered pyruvate metabolism in the contralateral normal-appearing brain parenchyma of patients with GBM, compared with healthy volunteers (participants vs volunteers: LP ratio, 0.33 ± 0.06 vs 0.23 ± 0.07, *P* = .009; BP ratio, 0.10 ± 0.03 vs 0.07 ± 0.04, *P* = .047).

## Introduction

Glioblastoma (GBM) is the most common and aggressive primary malignant brain tumor in adults, with a median survival of only 12–15 months despite aggressive therapy (1). This poor prognosis is partly due to its characteristic heterogeneity, which can be demonstrated morphologically and functionally by using conventional MRI. This heterogeneity also exists on a metabolic level, which results from a complex interplay between genomic and microenvironmental changes leading to metabolic reprogramming (2). This metabolic reprogramming may influence whether a GBM is predisposed toward infiltration or proliferation and to therapy resistance; highly proliferative cells downregulate glycolysis and upregulate the pentose phosphate pathway (3–5), and glioma stem cells are less glycolytic than differentiated cells, which may relate to radiation therapy resistance (6). Moreover, cellular and molecular heterogeneity (7) makes accurate phenotyping of patients difficult because of biopsy sampling error and is a key factor in therapeutic failure (8). Therefore, metabolic reprogramming of GBM represents an important target for novel therapeutic strategies (1,3), and noninvasive methods for imaging GBM metabolism could help to better characterize tumors and their early response to treatment (3,9).

MR spectroscopic imaging of hyperpolarized carbon 13 (^13^C)–labeled metabolites (hyperpolarized ^13^C MRI) is an emerging clinical tool for noninvasive assessment of metabolism in vivo (10). Metabolism of pyruvate, the product of glycolysis, has been widely studied by using this technique. Pyruvate lies at a metabolic crossroads, between conversion to lactate in the reaction catalyzed by cytosolic lactate dehydrogenase (LDH), and entry into the mitochondrial tricarboxylic acid cycle in the reaction catalyzed by pyruvate dehydrogenase (Fig E1 [supplement]). Pyruvate dehydrogenase transfers the pyruvate ^13^C label to carbon dioxide, which is in near equilibrium with bicarbonate, and the latter is detected because of its greater abundance at physiologic pH (10,11). Preclinical studies have shown increased lactate labeling in orthotopic GBM models and have demonstrated changes in lactate labeling following therapy (12–16). A study of orthotopically implanted, patient-derived xenograft models of GBM demonstrated a high degree of variability in lactate labeling between tumors, which could be explained by differences in the levels of the transcription factor c-Myc driving LDH-A expression and glycolytic activity (17). In humans, intravenous hyperpolarized [1-^13^C]pyruvate has been shown to result in both lactate and bicarbonate labeling in the healthy human brain, allowing assessment of both glycolytic metabolism in the cytosol and oxidative metabolism in the mitochondria (18). Previous clinical studies using ^13^C MRI with hyperpolarized [1-^13^C]pyruvate have demonstrated the feasibility of imaging GBM metabolism in patients across a spectrum of clinical presentations, all performed following some form of treatment (19–23). Moreover, a correlation between gene expression and lactate labeling has not been demonstrated previously (19–25). In this exploratory study, we have characterized hyperpolarized [1-^13^C]pyruvate metabolism in participants with treatment-naive isocitrate dehydrogenase wild-type GBM by using hyperpolarized ^13^C MRI. The metabolic images were compared with conventional contrast agent–enhanced proton MR images.

## Materials and Methods

### Participant Selection, Enrollment, and Clinical Monitoring

This prospective study was approved by a regional research ethics committee (reference 16/EE/0184). Between November 2016 and October 2018, eight consecutive treatment-naive participants (six men, two women; age [mean ± SD], 60 years ± 11; Table 1, Fig E2 [supplement]) with a presumed diagnosis of GBM scheduled for image-guided resection at our institution were imaged by using hyperpolarized ^13^C MRI after providing written informed consent. Exclusion criteria were clinical or imaging features that would suggest a secondary lesion, general contraindications for MRI, and age younger than 18 years. Seven participants were imaged successfully and included in this study. Imaging of one participant was abandoned because of failure of the acquisition protocol. Participant data were compared with previously published data from four healthy volunteers (18), the details of which are provided in Appendix E1 (supplement).

**Table 1:**
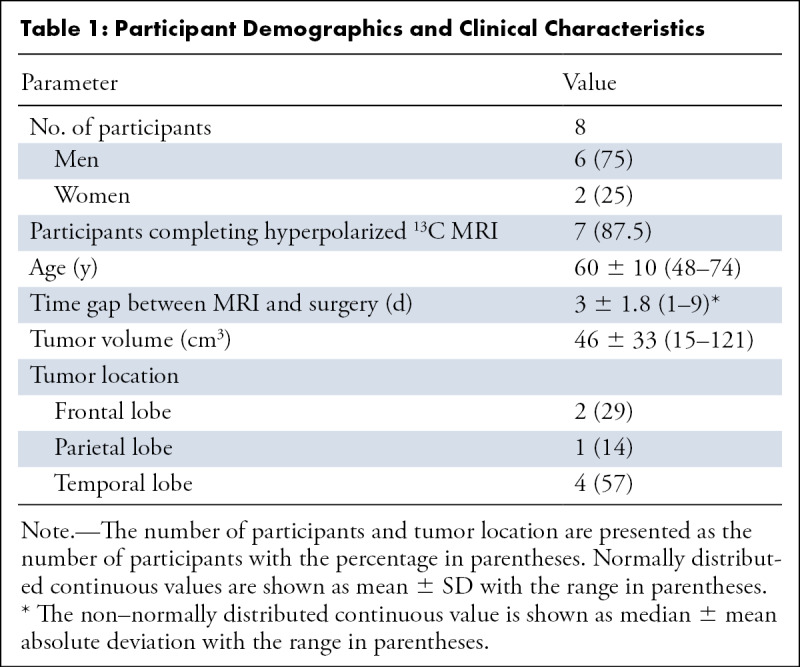
Participant Demographics and Clinical Characteristics

### Pyruvate Preparation and Hyperpolarization

Hyperpolarized [1-^13^C]pyruvic acid was prepared, and quality control checks were performed, as described in Appendix E1 (supplement). A volume of 0.4 mL/kg of approximately 250 mM hyperpolarized [1-^13^C]pyruvate was injected at 5 mL/sec by using an automatic MRI injection system (Spectris Solaris; MEDRAD), followed by 25 mL of a saline flush.

### Hydrogen 1 MRI and Hyperpolarized ^13^C MRI Acquisitions

MRI examinations were performed with a 3-T clinical imager (Discovery MR750; GE Healthcare) by using a dual-tuned ^13^C–hydrogen 1 (^1^H) quadrature transmit-receive head coil (Rapid Biomedical) and a 12-channel ^1^H head coil (GE Healthcare). The homogeneous coil sensitivity profile across the field of view allowed accurate quantification of brain metabolism and comparison between participants (18).

The hyperpolarized ^13^C MRI acquisition was obtained by using a dynamic, iterative decomposition with echo asymmetry and least-squares estimation spiral chemical shift imaging sequence: field of view, 240 × 240 mm; repetition time, 500 msec; echo time, 1.4 msec; flip angle, 15°; acquisition matrix, 40 × 40; reconstruction matrix, 128 × 128 (Fig E3 [supplement]); section thickness, 30 mm (26). Images were acquired every 4 seconds for a total of 60 seconds. Details of the ^1^H MRI acquisition are provided in Appendix E1 (supplement).

### Image Processing and Analysis

Image processing was performed in MATLAB (MATLAB 2017a, MathWorks) to provide the sum of the metabolite signals over the time course, to estimate the noise-corrected signal ratio maps (lactate-to-pyruvate [LP], bicarbonate-to-pyruvate [BP], and bicarbonate-to-lactate ratios), and to determine the apparent rate constant describing the exchange of label between pyruvate and lactate (*k*_PL_) and the apparent first-order rate constant describing conversion of pyruvate to bicarbonate (*k*_PB_), as described previously (18,27); further details are provided in Appendix E1 (supplement). Mean values for the summed metabolites in each region of interest (ROI) were referenced to the peak pyruvate signal intensity in each participant to allow group comparisons. For the histogram analysis, the tumor pyruvate, lactate, and bicarbonate signal intensities were normalized to the mean of the contralateral normal-appearing brain parenchyma (NABP) on the same axial imaging section to allow a direct comparison of heterogeneity within and between lesions.

A neuroradiologist (F.Z., with 9 years of experience in neuroimaging) outlined the ROIs on the non–contrast-enhanced ^1^H three-dimensional T1-weighted fast spoiled gradient-echo acquisition images by using OsiriX (version.8.5.2, Pixmeo SARL). The images were acquired with the dual-tuned ^13^C-^1^H coil to ensure accurate co-registration with the metabolite maps. Fluid-attenuated inversion recovery images and postcontrast ^1^H three-dimensional T1-weighted fast spoiled gradient-echo acquisition images were also used to reference these ROIs. Further details are provided in Appendix E1 (supplement). ROIs were positioned to exclude major vessels, where possible, to reduce bias in the analysis. Metabolism in the NABP was assessed in two ways: in the entire hemispheres (excluding the tumor on the ipsilateral side, with a margin to avoid the peritumoral fluid-attenuated inversion recovery hyperintensity) and in an ROI in the contralateral hemisphere that was the mirror image of the tumor ROI in the ipsilateral hemisphere.

### Histopathologic, Immunohistochemical, and Western Blot Analyses

Imaging was compared with immunohistochemical (IHC) and Western blot data obtained from multiregional biopsy samples. Multiple biopsy samples obtained from each patient were targeted to regions of high and low metabolism on the hyperpolarized ^13^C MR images (average of 5.7 ± 1.9 per patient; range, 2–8). Regions with high or low lactate labeling were labeled with 1-cm^2^ circular ROIs on the three-dimensional T1-weighted images and were targeted for biopsy. The sampling plan was discussed with the operating neurosurgeon prior to surgery, and a member of the research team was present during surgery to assist with targeted sampling, collection, handling, and freezing of the samples. Details of IHC and Western blot analysis are provided in Appendix E1 (supplement). The LP ratio from each ROI was compared with LDH-A expression in the targeted biopsy sample from that site. The expression of carbonic anhydrase IX (CAIX) as a marker of hypoxia (28); monocarboxylate transporter 1 (MCT1), the membrane transporter responsible for pyruvate uptake; and MCT4, the membrane transporter largely responsible for lactate and ketone body export (29), were determined by using IHC analysis and were compared with the ^13^C imaging data.

### Statistical Analysis

Statistical analysis was performed by using MATLAB (MATLAB and Machine Learning Toolbox, MATLAB 2017a; MathWorks), SPSS (version 18.0, SPSS), and RStudio (version 1.1.463 for Macintosh, RStudio), which is based on R version 3.5.1 (R Foundation for Statistical Computing platform [30]). The Shapiro-Wilk test was used to test for normality. Subsequently, a log_10_ transformation was applied to the data that were originally considered to be normally distributed, and the normality of data distribution was confirmed by using the Shapiro-Wilk test and the Jarque-Bera normality test. Continuous data were expressed as mean ± SD (minimum − maximum) for normally distributed data and as median ± median absolute deviation (minimum − maximum) for non–normally distributed data.

The two-tailed paired *t* test, unpaired *t* test, and Wilcoxon signed rank test were used to compare the metabolites, ratios, and exchange rate constants derived from the tumors and NABP as appropriate, depending on the distribution of the data. Paired and unpaired *t* tests were performed, and the *t* values, *df*, and *P* values are presented as follows: *t*[*df*], *P*. For unpaired *t* tests, we assumed unequal variance and applied the Welsh *df* modification. Correlations among metabolites, LDH-A expression, and IHC markers were determined by using the Spearman ρ correlation coefficient. For tissue samples, *P* values were derived by assuming independence between samples (8). Correlations between volumes and LP and BP ratios were determined by using regression analysis.

To determine the statistical significance of all the test results, a base set *P* value of .05 was chosen. Subsequently, significance was corrected for multiple comparisons by using the Benjamini-Hochberg procedure and computing the false discovery rate at 5%. Raw *P* values are shown, and significance is stated according to the Benjamini-Hochberg procedure.

## Results

### Quality Control Measurements

The average [1-^13^C]pyruvate polarization was 22% ± 4 (range, 16.3%–28.0%); the pyruvate concentration was 256 mM ±12 (232 –268 mM), and the pH was 7.7 ± 0.2 (range, 7.3–8.0). The time delay between dissolution and pyruvate injection was 59 seconds ± 4 (range, 54–65 seconds) (Table E1 [supplement]).

### Tumor Metabolism Measured with Hyperpolarized ^13^C MRI

**Figure 1:**
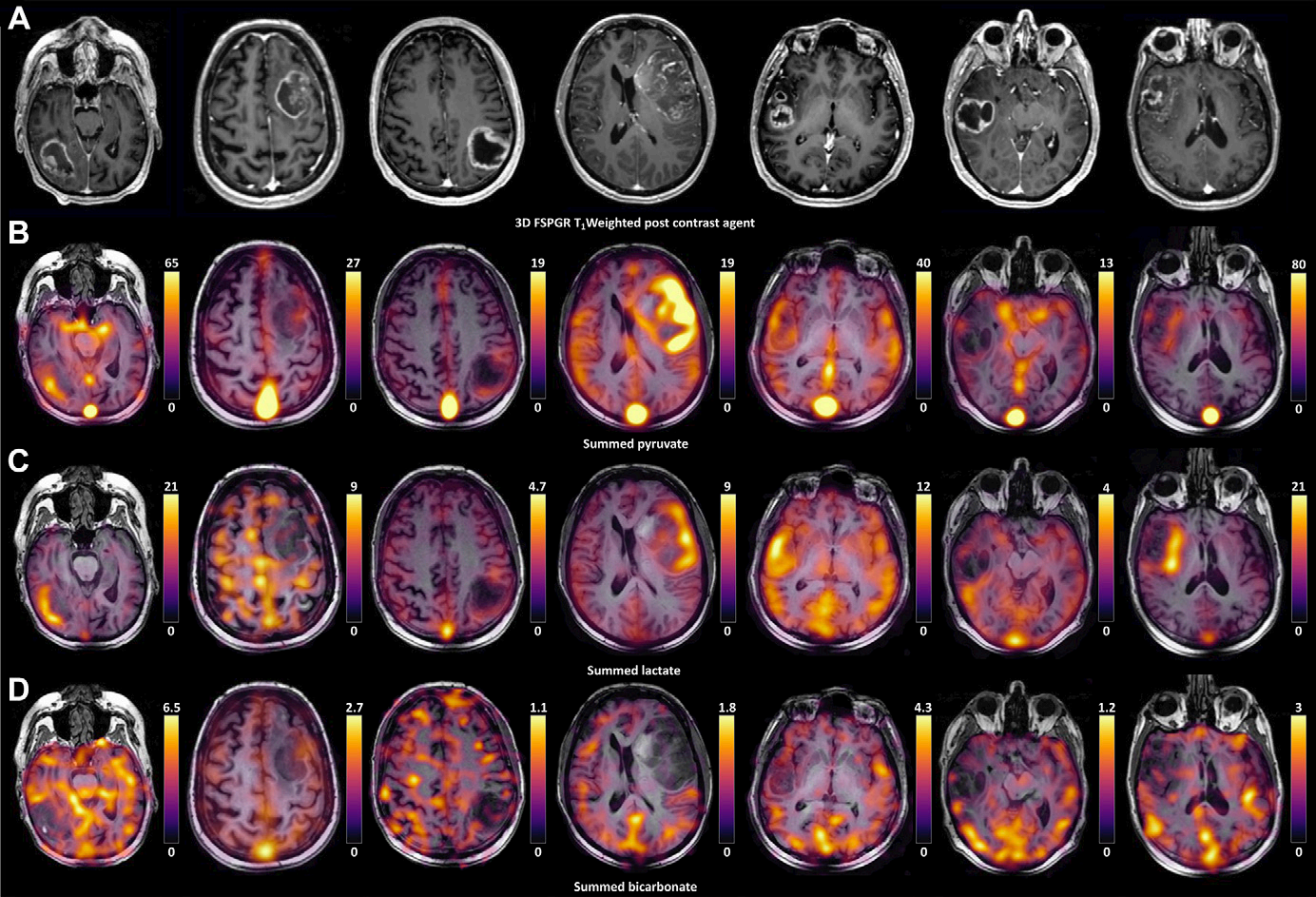
Hyperpolarized ^13^C MR images from all seven patients. **(A)** Grayscale axial contrast-enhanced ^1^H three-dimensional (3D) T1-weighted fast spoiled gradient-echo (FSPGR) images through the center of the lesion for each patient and the corresponding unenhanced images overlaid with the **(B)** pyruvate, **(C)** lactate, and **(D)** bicarbonate color maps summed over the time course.

**Figure 2:**
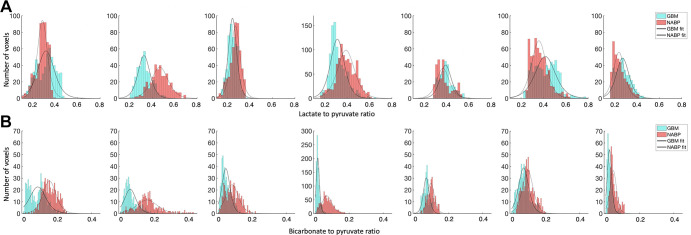
Histograms of the **(A)** lactate-to-pyruvate and **(B)** bicarbonate-to-pyruvate ratios in each voxel from the section through the center of the lesion for each patient (*n* = 7) with an overlying polynomial fit; glioblastoma (GBM) data are shown in blue, and the normal-appearing brain parenchyma (NABP) data are shown in red.

Hyperpolarized [1-^13^C]pyruvate, [1-^13^C]lactate, and [^13^C]bicarbonate signals were observed in all seven isocitrate dehydrogenase wild-type tumors, as well as in normal-appearing brain, following injection of hyperpolarized [1-^13^C]pyruvate (Fig 1). Figure 2 shows the LP and BP ratios calculated from these summed signals. The metrics derived from these data are summarized in Table 2, and data for each individual patient are shown in Table E2 (supplement).

**Table 2:**
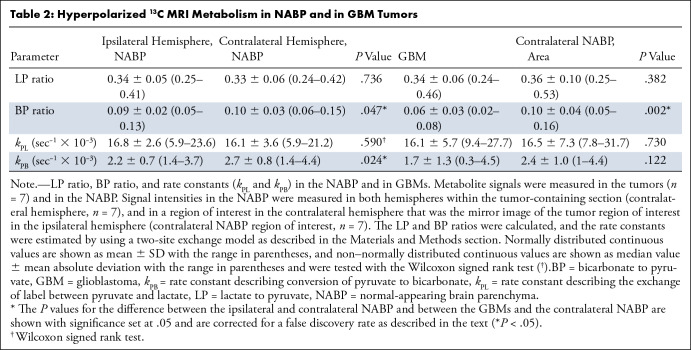
Hyperpolarized ^13^C MRI Metabolism in NABP and in GBM Tumors

**Figure 3:**
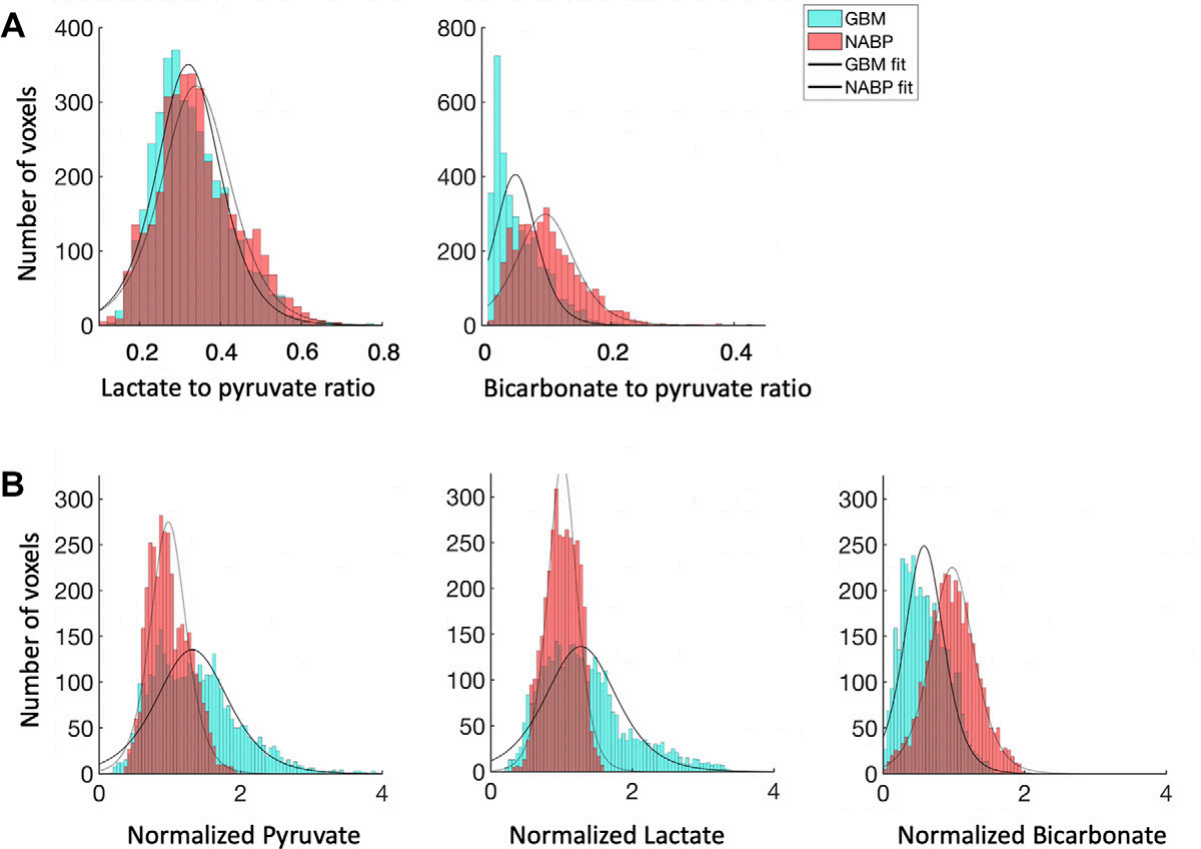
Average labeled metabolite distribution for the entire patient cohort (*n* = 7). Histograms show the **(A)** average lactate-to-pyruvate and bicarbonate-to-pyruvate ratios and **(B)** normalized signal intensities for pyruvate, lactate, and bicarbonate with an overlying polynomial fit. Normalization was performed relative to the normal-appearing brain parenchyma (NABP). Ratios for glioblastoma (GBM) are shown in blue, and ratios for NABP are shown in red.

The summed pyruvate and lactate signal intensities were significantly higher in tumors than in the contralateral NABP (*n* = 7, *t*[6] = 3.6, *P* = .01, and *t*[6] = 3.3, *P* = .02, respectively). However, we found no evidence of differences in the median *k*_PL_ (*n* = 7, *t*[6] = −0.36, *P* = .73) or the LP ratio (*n* = 7, *t*[6] = −0.94, *P* = .38) between the tumor ROIs and the contralateral NABP. The LP showed a high degree of intralesional and interpatient heterogeneity (Fig 2) and a wide variation in the LP ratio between participants with GBM. Specifically, some tumors demonstrated an LP ratio higher than that in the NABP (participants 1 and 6), whereas others showed a lower ratio. However, there was a consistent reduction in the tumor bicarbonate and BP ratio compared with those in the NABP (bicarbonate: *n* = 7, *t*[6] = −5.27, *P* = .002; BP ratio: *n* = 7, *t*[6] = −5.14, *P* = .002; Figs 2, 3).

Analysis of the summed lactate and pyruvate signal intensities in the individual participant tumors showed a strong positive correlation (ρ = 0.92, *P* < .001; Fig E4 [supplement]). The same was true for bicarbonate labeling, with a positive correlation between the summed pyruvate and bicarbonate signal intensities being shown (ρ = 0.66, *P* < .001; Fig E4 [supplement]).

### Metabolism of the NABP

The pyruvate and lactate signal intensities were higher in the ipsilateral NABP than in the NABP in the contralateral non–tumor-bearing hemisphere, although this did not reach statistical significance (*n* = 7, *P* = .26 and *P* = .24). There was no evidence of differences in the LP ratio (*n* = 7, *t*[6] = 0.35, *P* = .74) or the *k*_PL_ (*n* = 7, *P* = .59) between the ipsilateral and contralateral NABP. The summed bicarbonate signal intensity (*n* = 7, *t*[6] = −2.95, *P* = .03), BP ratio (*n* = 7, *t*[6] = −2.49, *P* = .047), and *k*_PB_ (*n* = 7, *t*[6] = −3.1, *P* = .02) were all significantly lower in the NABP in the ipsilateral hemisphere than in the NABP in the contralateral hemisphere.

In the healthy volunteers, the whole-brain average LP ratio determined from data acquired contemporaneously with the data shown here was 0.23 ± 0.07 (18), which is lower than in the GBM cohort (healthy volunteers: *n* = 4, participants with GBM: *n* = 7; *P* = .009). Similarly, the whole-brain average BP ratio was lower in healthy volunteers (0.07 ± 0.04) than in participants with GBM (healthy volunteers: *n* = 4, participants with GBM: *n* = 7; *P* = .047) (18).

### ^1^H MRI Measurements of Tumor Volume

**Figure 4:**
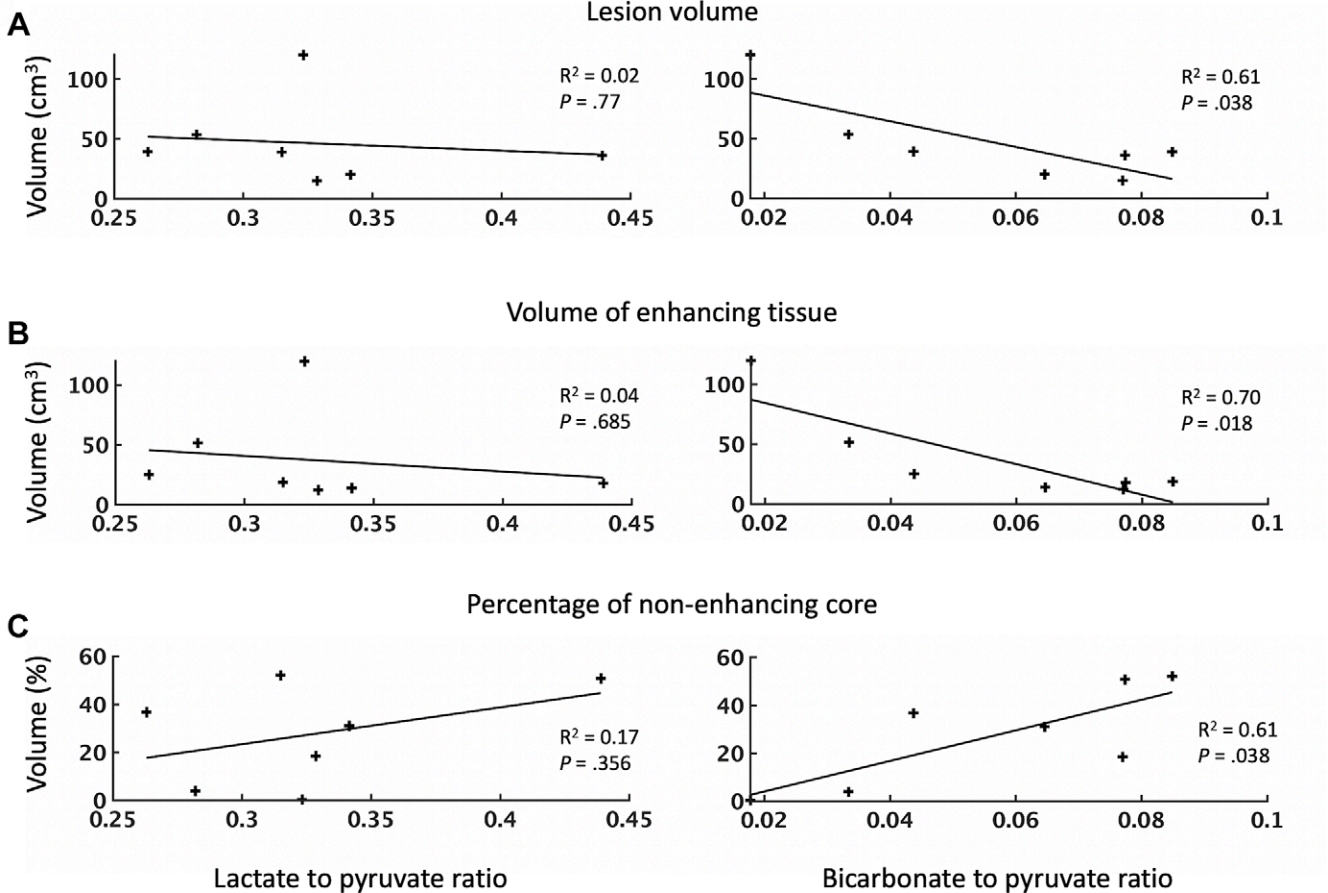
Dependence of metabolite signal ratios (lactate-to-pyruvate and bicarbonate-to-pyruvate ratios) on **(A)** tumor volume, **(B)** volume of enhancing tissue, and **(C)** percentage of nonenhancing tumor core. Each point represents an individual participant. The lesion volume and the volume of enhancing tissue are expressed in centimeters cubed; the nonenhancing core is expressed as a percentage of the entire lesion volume. The *R*^2^ values, representing the goodness of each fit, and the corresponding *P* values for each regression are given. The level of significance was set at .05.

The relationship between tumor volume, as measured by contrast-enhanced ^1^H three-dimensional T1-weighted imaging, and the LP and BP ratios is shown in Figure 4. The average volume of the lesions was 46 cm^3^ ± 33 (range, 15–121 cm^3^) with a 28% ± 19 (range, 0.4%–52%) nonenhancing core. Regression analysis demonstrated no significant correlation between the LP ratio and the total tumor volume, enhancing volume, or percentage of nonenhancing core (*n* = 7; *P* = .77, .68, and .36, respectively). In contrast, the BP ratio showed a significant decrease with increasing lesion volume (*n* = 7, *R*^2^ = 0.61, *P* = .04) and enhancing volume (*n* = 7, *R*^2^ = 0.70, *P* = .02) and conversely increased with an increasing percentage of nonenhancing core (*n* = 7, *R*^2^ = 0.61, *P* = .04). Pyruvate and lactate demonstrated a weak negative correlation with the nonenhancing core that did not reach statistical significance (*n* = 7; *P* = .17 and *P* = .43, respectively).

### Correlations among Tumor Lactate Labeling, LDH-A Expression, and IHC Markers

**Figure 5:**
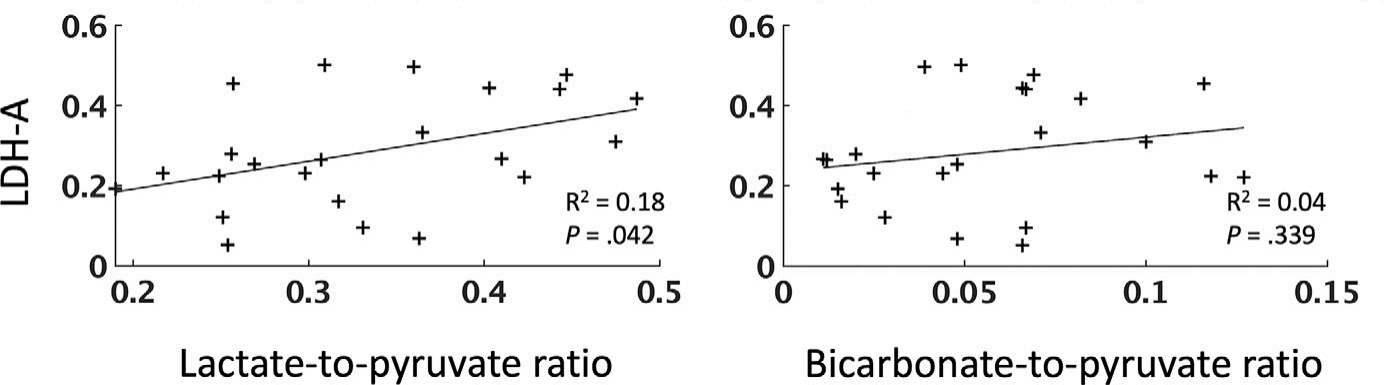
Relationship between lactate dehydrogenase A (LDH-A) expression and labeling of lactate and pyruvate following injection of hyperpolarized [1-^13^C]pyruvate. Scatterplots show the relationship between LDH-A expression and the lactate-to-pyruvate and bicarbonate-to-pyruvate ratios. Each point represents a tissue sample. The *R*^2^ values, representing the goodness of fit, and *P* values for each regression are shown.

**Figure 6:**
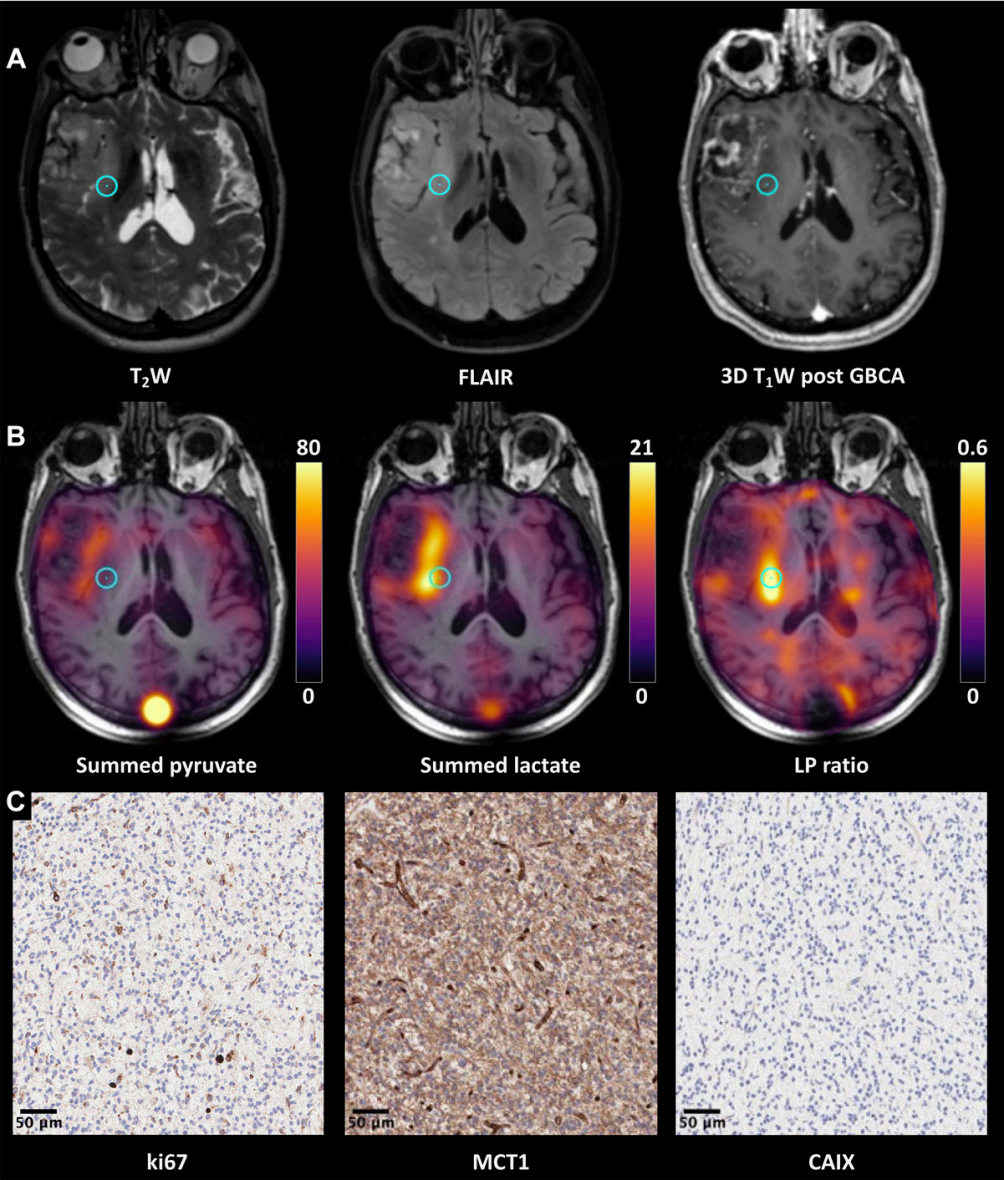
**(A–C)** Proton images, hyperpolarized ^13^C MR images, and immunohistochemical (IHC) data from participant 7 (74-year-old man with glioblastoma). **(A)** Grayscale axial three-dimensional (3D) T2-weighted (T_2_W), fluid-attenuated inversion recovery (FLAIR), and gadolinium-based contrast agent (GBCA)–enhanced 3D T1-weighted (T_1_W) fast spoiled gradient-echo images through the center of the lesion. There is a lesion within the right anterior temporal lobe demonstrating T2-weighted and FLAIR hyperintensity involving the right insula and external capsule and reaching the lentiform nucleus. **(B)** The corresponding pyruvate and lactate maps summed over the entire time course and the lactate-to-pyruvate (LP) ratio map are shown in color superimposed on the T1-weighted images before contrast enhancement. The metabolic maps reveal heterogeneity, with higher pyruvate and lactate being shown in the medial aspect of the lesion; the LP ratio was particularly higher in the posterior part of insula. **(C)** Representative IHC imaging, shown with a 20× magnification, from the target region of interest highlighted on the ^1^H and ^13^C MR images (blue circle) stained for ki-67, monocarboxylate transporter 1 (MCT1), and carbonic anhydrase IX (CAIX). Details on IHC analysis are provided in Appendix E1 (supplement); in brief, the antibodies used for staining were: M7240 for ki-67, HPA003324 for MCT1, and NCL-L-CAIX for CAIX. Histopathologic findings demonstrated a homogeneous high-grade tumor with MIB-1 staining of approximately 8%, high MCT-1 staining, and no significant staining for CAIX.

The concentration of LDH-A in tumor biopsy samples, quantified by using Western blotting, exhibited a moderate positive correlation with the local LP ratio (*n* = 24, ρ = 0.43, *P* = .04; Fig 5, Table E3 [supplement]). CAIX showed a moderate negative correlation with summed pyruvate (*n* = 29, ρ = −0.59, *P* < .001) and summed lactate (*n* = 29, ρ = −0.54, *P* < .001). We found no evidence of a correlation between MCT1 and any of the measured ^13^C metrics, including the sum of pyruvate, lactate, and bicarbonate signal intensities (*n* = 29, all *P* values > .11). MCT4 was positively correlated only with the BP and bicarbonate-to-lactate ratios (*n* = 29, ρ = 0.4, *P* = .03, and ρ= 0.4, *P* = .03, respectively). Figure 6 shows a representative example demonstrating the correlation among proton images, metabolite maps, and IHC for Ki67, MCT1, and CAIX obtained from the region highlighted in the images.

## Discussion

In this prospective study, we evaluated the hyperpolarized ^13^C MRI technique in participants with treatment-naive primary GBM and have correlated lactate labeling with tissue obtained at surgery. To our knowledge, large studies have not yet been performed that investigate the use of hyperpolarized ^13^C MRI within a similar cohort, and this small cohort therefore provides important data on changes in metabolism in GBM and the surrounding normal brain in treatment-naive patients. We found that the LP and BP signal ratios demonstrated significant intralesional and interpatient heterogeneity, although there was no evidence of a difference in the average LP ratio or *k*_PL_ between tumors and the contralateral NABP. The higher pyruvate and lactate labeling in tumors compared with the contralateral NABP may reflect increased pyruvate delivery, given the strong correlation between pyruvate and lactate signal intensities, implying that lactate labeling is partly determined by pyruvate delivery. The negative correlation between pyruvate and CAIX suggests poor delivery of pyruvate in hypoxic regions of the tumor, which is also supported by the weak inverse relationship between the percentages of tumor necrosis and both pyruvate and lactate. This heterogeneity in lactate signal has also been observed in patient-derived xenograft models of GBM (17) and in the few clinical cases published to date (19,23). In contrast, the BP ratio was consistently lower in tumors than in the NABP, implying that mitochondrial metabolism is impaired.

A previous ^1^H MRI study of patients with GBM and healthy volunteers using chemical exchange saturation transfer measurements showed metabolic changes in the NABP contralateral to the tumor (31). Comparing the LP ratios in the NABP measured here with those reported previously by using identical methods in healthy volunteers (18), we found significantly higher LP and BP ratios in the NABP of the tumor-bearing brains. Although partial volume effects could influence the measured LP ratios of the NABP in the contralateral hemisphere, taken together with the previously published chemical exchange saturation transfer results, these data imply that the presence of GBM alters both oxidative and reductive metabolism of the whole brain. Partial volume effects cannot explain the increase in the BP ratio in the NABP compared with the healthy brain area, as inadvertent inclusion of tumor would decrease and not increase the measured ratio compared with its true value. However, these results need to be validated in larger cohorts in the future.

The exchange of hyperpolarized ^13^C label between the injected pyruvate and endogenous lactate pools depends on a number of factors: pyruvate delivery, expression of the pyruvate transporters (MCTs), and LDH activity, which catalyzes the exchange of ^13^C label between pyruvate and lactate (10). In the GBM tumors studied here, there was no correlation between the total tumor volume, enhancing volume, or nonenhancing core and the LP ratio. This is in contrast to a previous study in patients with breast cancer, in which the LP ratio was increased in larger tumors (27). In the GBM tumors studied here, there was a correlation between the LP ratio and tumor LDH-A expression but no correlation between the LP ratio and MCT1 expression. This suggests that in GBM, increased lactate labeling is driven primarily by increased pyruvate delivery and LDH-A expression. A previous study in GBM patient–derived xenograft models implanted orthotopically in the rat observed a correlation between lactate labeling and expression of c-Myc, LDH-A, hexokinase II, MCT1, and MCT4 (17).

Detecting metabolic heterogeneity has implications for treatment of patients with GBM, including tailoring therapy or the radiation therapy dose. Intratumoral variations in lactate labeling could be used to derive metabolic habitats (32) or to guide biopsies. Metabolic maps may also be useful for detecting early response to chemotherapy and radiation therapy (16). For instance, hyperpolarized ^13^C MRI could be used to assess changes in metabolism with isocitrate dehydrogenase inhibition, which has shown promising results in vitro and in animal models (33).

This study had several limitations. First, although our sample size is larger than has been investigated in previous studies, it remains small and warrants further work to investigate the origin of intertumoral metabolic heterogeneity. A technical limitation of this study was the relatively low spatial resolution, which prevented assessment of the peritumoral environment and comparison with conventional ^1^H sequences. The ability to distinguish tumor infiltration and peritumoral edema depends on the relative volume of tumor cells in each voxel compared with normal tissue, as well as the relative difference in metabolism between the two. Improvements in spatial resolution will enable evaluation of the peritumoral region in future studies. Additionally, although there was a statistically significant difference in the BP ratio between the GBMs and NABP, a further technical limitation of the study was low bicarbonate signal compared with that of pyruvate and lactate. A final limitation was the inability to compare imaging, IHC, and Western blot data in healthy tissue samples; these correlations may be explored in animal models to corroborate the findings from human imaging in the future.

In conclusion, this study showed variation in the levels of lactate labeling in GBM, both within and between tumors, whereas bicarbonate labeling was consistently lower in tumors when compared with the surrounding NABP. The differences in lactate labeling may be explained by differences in pyruvate delivery to the tumor and LDH-A expression. The LP and BP ratios in the hemisphere contralateral to the tumor were higher than in the brains of healthy volunteers, suggesting that the presence of a GBM in the brain increases both glycolytic and oxidative activity in the NABP. We have revealed insights into the effect of the tumor on normal-appearing brain metabolism. These results will have important implications for how this technique can be applied in future larger studies and has provided a biological explanation for why lactate labeling varies between and within these tumors.
